# Trends and Outcomes of Naloxone Use for Iatrogenic Opioid Overdose in Patients on General Wards in a Swiss Hospital: A Ten-Year Retrospective Case Series Analysis

**DOI:** 10.2147/DHPS.S607184

**Published:** 2026-05-13

**Authors:** Maurice Würsten, Carla Meyer-Massetti, Anne Leuppi-Taegtmeyer, Aljoscha N Goetschi

**Affiliations:** 1 Clinical Pharmacology and Toxicology, Department of General Internal Medicine, University Hospital of Bern, Bern, 3010, Switzerland; 2 Institute of Primary Health Care (BIHAM), University of Bern, Bern, 3010, Switzerland; 3 Department of Patient Safety, University Hospital of Basel, Basel, 4031, Switzerland; 4 Department of Clinical Research, University of Basel, Basel, Switzerland

**Keywords:** opioids, medication errors, overdose, patient safety

## Abstract

**Introduction:**

Opioids carry an inherent high risk of adverse drug events (ADEs), although these are generally predictable and avoidable through proper management and monitoring. Naloxone, an opioid antagonist, is used to counteract severe opioid overdoses.

**Aim:**

This study aimed to estimate the incidence of iatrogenic opioid overdoses and describe prescribing patterns before, during and after them.

**Methods:**

We conducted a ten-year retrospective case series analysis of naloxone use among inpatients at a large, multisite university hospital in Switzerland from January 2014 to December 2023. Cases were included if an adult patient had received naloxone after opioid administration and overdose. We excluded patients who received naloxone during surgery or in intensive care units.

**Results:**

Of the 671 uses of naloxone identified, 121 (18.0%) met our inclusion criteria. Yearly naloxone use incidence was 11.0 per 10,000 inpatients, increasing slightly from 2014 to 2023. The median daily opioid dose requiring naloxone was 73 morphine milligram equivalents (MME). According to the Schumock and Thornton scale criteria, we considered 82 (67.8%) of the 121 opioid overdoses to have been potentially preventable The median opioid doses received before hospital admission (29.4 MME) were higher than at discharge (15.0 MME). Nevertheless, 71 (68.3%) of 104 discharged patients were still prescribed at least one opioid.

**Conclusion:**

This study found that iatrogenic opioid overdoses were relatively uncommon. However, the considerable number of preventable opioid overdoses estimated leads us to conclude that opioid stewardship programmes should be recommended across Switzerland.

## Introduction

Opioids are analgesic medications used to treat moderate to severe pain. Although they are important therapeutic options in multimodal pain management strategies, they carry an inherent high risk of adverse drug events (ADEs).^1^,^2^ Proper management and monitoring generally make these ADEs predictable and avoidable.^3^ The most severe ADEs associated with opioids include respiratory depression, loss of consciousness, stupor and hypothermia.^2^ Naloxone, an opioid antagonist, is used to counteract severe opioid overdoses.^1^ It can be administered intravenously, intramuscularly, subcutaneously or nasally. Intranasal naloxone’s bioavailability is 50%, and its rapid onset of action ranges from one to a few minutes.^4^ The standard intravenous naloxone dose for patients without a known opioid dependency is 0.4 mg,^4^ and peak opioid receptor blockade occurs 25 minutes after administration.^5^ Some patients may experience opioid withdrawal after receiving naloxone, but due to its short half-life, the symptoms are usually short-lived. While they are uncomfortable for the patient, they are rarely life-threatening.^6^,^7^

The consequences of a life-threatening opioid overdose are severe and multifaceted. Patients not only face the immediate dangers associated with overdose but also endure longer hospital stays, increased risks of readmission and significantly higher healthcare costs.^8–10^ Extended stays and readmissions place substantial burdens on patients and healthcare systems, highlighting the need for preventative measures to mitigate the risk of opioid overdoses.

There is limited information about the incidence of naloxone use to treat iatrogenic opioid overdoses in general inpatient populations. Most evidence concerns surgical patients, especially in postoperative settings. A 2024 review identified eight studies reporting mean naloxone use incidences of 3.65–53.28 per 10,000 postoperative inpatients.^11^ Less evidence is available for medical patients, but one study reported an incidence of naloxone use of 11.5 per 10,000 inpatients.^12^ One 12-hospital study calculated a naloxone use incidence of 108.5 per 10,000 inpatients receiving opioids (158 cases among 14,558 patients on opioids).^13^ Thus, there is uncertainty about the incidence of naloxone use among general patient populations in hospitals.

Furthermore, debates on the risk factors for opioid overdoses are ongoing. Some authors have reported associations with particular disorders, including obstructive sleep apnoea and other respiratory, renal and cardiac diseases.^14^,^15^ Others have reported associations with higher opioid doses or other sedative comedication.^13–16^ Older age and obesity have also been reported as potential risk factors for naloxone administration.^17^ Despite studies reporting on these risk factors, more information is needed on the populations receiving naloxone in hospitals.

Information on iatrogenic opioid overdoses among inpatients in Switzerland is also limited, and this is especially concerning because opioid prescriptions, particularly for potent opioids, have been rising nationally.^18^ Inpatients may therefore be at an increased risk of iatrogenic opioid overdose. This issue needed to be assessed and addressed.

The present study thus analysed the incidence of iatrogenic opioid overdoses and subsequent treatment using naloxone among the inpatients at a large, multisite university hospital in Switzerland. By examining these cases, the study aimed to: 1) describe the population requiring naloxone to treat opioid overdoses; 2) estimate the incidence of these overdoses; 3) describe prescribing patterns before, during and after opioid overdoses; 4) estimate the number of potentially preventable opioid overdoses; and 5) detail the hospitalisation outcomes after opioid overdoses.

## Methods

### Study Design

We conducted a ten-year retrospective case series analysis of naloxone treatments for iatrogenic opioid overdoses from 2014–2023 at the Insel Group—an integrated healthcare network including the University Hospital of Bern, Bern Rehabilitation Centre, and three other local regional hospitals in Switzerland, together treating approximately 55,000 inpatients yearly. This study was approved by the Ethics Commission of the Canton of Bern (project-ID: 2024–01506) and complies with the Declaration of Helsinki. All included patients had signed a waiver allowing the further use of their data. We followed the Strengthening the Reporting of Observational Studies in Epidemiology guidelines.^19^

### Patients

We examined the cases of adult inpatients hospitalised in the Insel Group between 1 January 2014 and 31 December 2023, and we included all those who had received opioids (anatomical therapeutic chemical [ATC] codes: N02A*) and subsequently naloxone (ATC: V03AB15). Fixed combinations of opioids and naloxone, where naloxone was primarily included to prevent constipation, were counted as opioid treatments. Importantly, naloxone use is a well-established indicator of potential severe opioid-induced ADEs, showing high specificity and sensitivity.^20^ We only included patients who had provided written consent to the further use of their data.

We defined an iatrogenic opioid overdose as naloxone administration within 24 hours of receiving opioids, accompanied by a subsequent documented improvement in symptoms. This could mean less sedation, a faster respiratory rate or better oxygen saturation. Eligibility was assessed through a review of medical records. We excluded patients receiving naloxone during surgery and in intensive or intermediate care units because naloxone is used in standard anaesthesia to reverse sedation and because their data were unavailable to us for research purposes.

### Variables and Data Management

The list of adult patients administered opioids followed by naloxone came from our in-hospital data science centre, and we then verified the additional eligibility criteria. Eligible patients were assigned an anonymous patient identifier, and the required data variables were extracted into REDCap^®^ software.

We manually extracted variables from electronic patient charts in the ipdos^®^ clinical information system (CGM CompuGroup Medical Schweiz AG, Version 7.21.1.5). Variables included patient characteristics, information on hospital admission, clinical parameters and medication use (eg. opioids used, opioid regimens) 24 hours before naloxone treatment, details of that naloxone use (eg. dose, dosing frequency, route of administration), outcomes following naloxone use and information on hospital discharge. Importantly, medication prescribed during a surgery was not considered, as we had incomplete access to these data. We also evaluated whether the opioid overdose would have been preventable according to the Schumock and Thornton criteria.^21^ These are a list of seven questions covering topics such as the clinical appropriateness of the drug used, dose appropriateness, route and frequency of the drug used, and potential drug–drug interactions. If one of the answers was unfavourable, eg. the drug was inappropriate for the patient’s clinical condition, then the ADE was considered potentially preventable.^21^ These criteria are widely used in ADE research.^22^,^23^

Data extraction was primarily performed by a single analyst (MW); a second analyst (ANG) verified a random sample of 25% of these. The 8% of cases involving discrepancies were resolved through discussion. The analysts independently judged preventability, and a consensus was reached through discussion.

### Statistical Methods

Our descriptive analyses used medians and interquartile ranges (IQRs) or percentages, where appropriate. For comparability, opioid doses were standardised to morphine milligram equivalents (MME) following the Centre for Disease Control and Prevention’s guidelines.^24^

We estimated the incidence of naloxone use per 10,000 inpatients and per 10,000 inpatients receiving opioids at Insel Group sites. We calculated 95% confidence intervals (95% CIs) using a gamma distribution.

We assumed that missing data were random, and we noted how many patients had available data at admission, during hospitalisation and at discharge.

## Results

**Figure 1:**
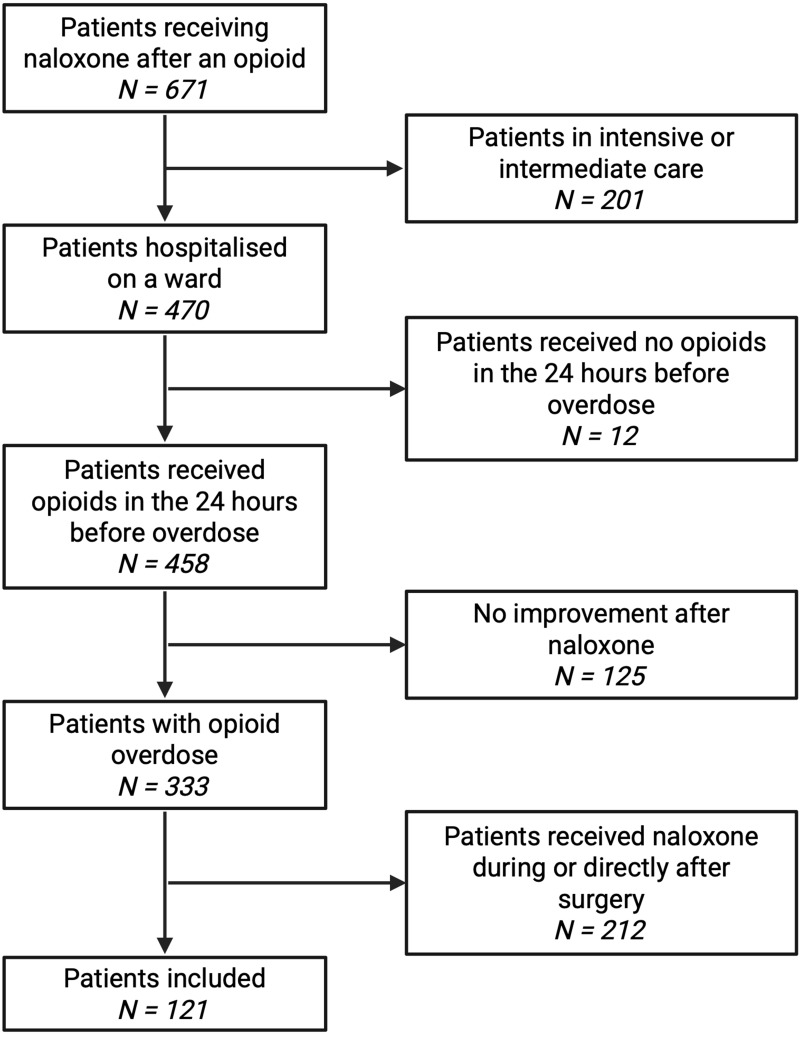
Study flowchart.

We identified 671 inpatients treated with naloxone after an opioid, across the entire ten-year study period, with 121 meeting all our inclusion criteria (see Figure 1) and more men (68, 56%) than women (53, 44%). The median age was 67 years (IQR: 56–75). Patients suffered from multiple chronic conditions, with a median of 12 diagnoses (7–15) (see Table 1 for more information).

**Table 1:** Overview of Study Population Characteristics

	N = 121^a^
Women	53 (44%)
Age, *years*	67 (56–75)
BMI, *kg/m^2^*	23.4 (20.4–28.5)
Length of hospital stay, *days*	20 (8–32)
Number of diagnoses	12 (7–15)
Psychiatric diseases	51 (42%)
CKD	46 (38%)
eGFR, *mL/min*	59 (32–101)
Frailty	33 (27%)
COPD	20 (17%)
HIV	3 (3%)
Asthma	2 (2%)

**Notes**: ^a^ categorical values use n (%); continuous variables use median (interquartile range [IQR]).

**Abbbreviations**: BMI, body mass index; CKD, chronic kidney disease; COPD, chronic obstructive pulmonary disease; eGFR, estimated glomerular filtration rate; HIV, human immunodeficiency virus.

### Incidence of Naloxone Use

**Figure 2:**
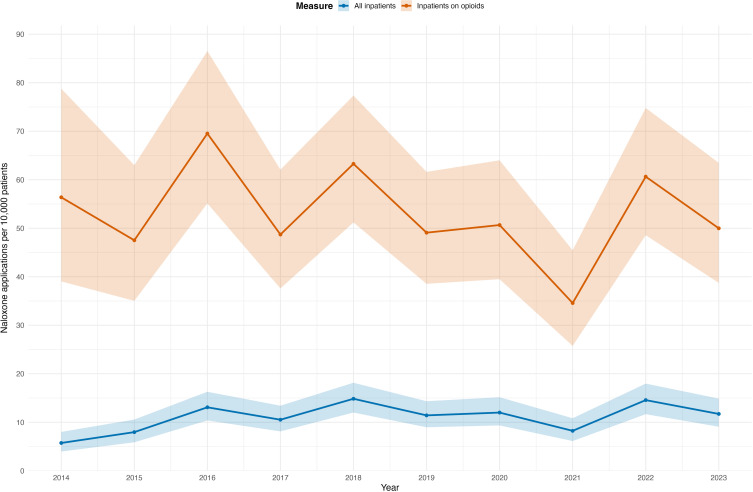
Trajectories of the yearly naloxone incidence rates per 10,000 inpatients (blue) and per 10,000 inpatients receiving opioids. Ribbons depict 95% confidence intervals. Cases per year, number of inpatients and number of inpatients on opioids can be seen in Supplementary Table 1.

We estimated an incidence of 11.0 naloxone treatments (10.2–11.9) per 10,000 inpatients and 52.7 naloxone treatments (48.8–56.8) per 10,000 inpatients receiving opioids over the entire study period. The trajectories of the yearly incidence rates are shown in Figure 2.

### Prescription Patterns Before, During and After Opioid Overdoses

Patients had received a median of 73 MME (28–178) in the 24 hours before their naloxone treatment, with a median of 60 MME (15–175) from their fixed dose opioid prescription and 7.5 MME (0–22.5) from their PRN prescription. They then received a median total dose of 0.4 mg (0.2–0.6) naloxone to treat their overdose. Most patients (67, 55%) received naloxone just once, 34 (28%) twice and 20 (17%) three times or more. Naloxone was administered intravenously to 112 patients (93%), subcutaneously to 5 (4%), and to 4 (3.3%) via a combination of routes.

Patients’ charts reported different symptoms of opioid overdose. Somnolence was the most frequent, with 74 patients affected (61%), followed by sopor/coma (46, 38%), hypoxia (42, 35%), miosis (41, 34%), hypotension (33, 27%), bradypnea (24, 20%) and respiratory arrest (17, 14%). Other symptoms, such as dysarthria, hypercapnia or respiratory acidosis, were less frequently reported.

Prescription patterns changed between hospital admission, the time of opioid overdose and hospital discharge. At the time of overdose, patients were generally taking more comedications (median = 14 [IQR: 11–19] vs 10 [IQR: 7–14] at admission or 13 [IQR: 10–16] at discharge). More patients were taking opioids at discharge (68%) than at admission (56%), but MMEs were lower (median = 15 [IQR: 0–60] at discharge vs 29.4 [IQR: 0–120] at admission). The number of sedative drugs taken at admission was lower than at the time of opioid overdose (median = 1 [IQR: 1–3] vs 4 [IQR: 3–5]), and the number remained higher at discharge (median = 2 [IQR: 2–4]). See Table 2 for more information.

**Table 2:** Overview of Prescription Pattern Changes from Hospital Admission to 24 Hours Before Overdose to Discharge

	Admission^a^ n = 66	Overdose^a^ n = 121	Discharge^a^ n = 104
No. of drugs	10 (7–14)	14 (11–19)	13 (10–16)
No. of PRN drugs	0 (0–1)	8 (5–10)	2 (1–5)
Opioids, *yes*	38 (56%)	121 (100%)	71 (68%)
Concurrent opioids, *yes*	0 (0%)	47 (39%)	15 (14%)
MME [mg]	29.4 (0–120)	60 (15–175)	15 (0–60)
MME PRN [mg]	NA	35 (18–75)	0 (0–31)
No. of sedatives	1 (1–3)	4 (3–5)	2 (1–4)
Benzodiazepines, *yes*	16 (24%)	66 (55%)	29 (28%)
Z-Drugs, *yes*	8 (12%)	15 (12%)	14 (13%)
Gabapentinoids, *yes*	14 (21%)	33 (27%)	32 (31%)
Anticholinergics, *yes*	12 (18%)	42 (35%)	35 (34%)

**Notes**: ^a^ categorical values are shown as n (%); continuous variables are shown as median (interquartile range [IQR]).

**Abbreviations**: MME, morphine milligram equivalents; PRN, *pro re nata* (medication as needed); MME PRN, maximum quantity of opioids that the patient could have taken, not the actual PRN of opioids administered.

**Figure 3:**
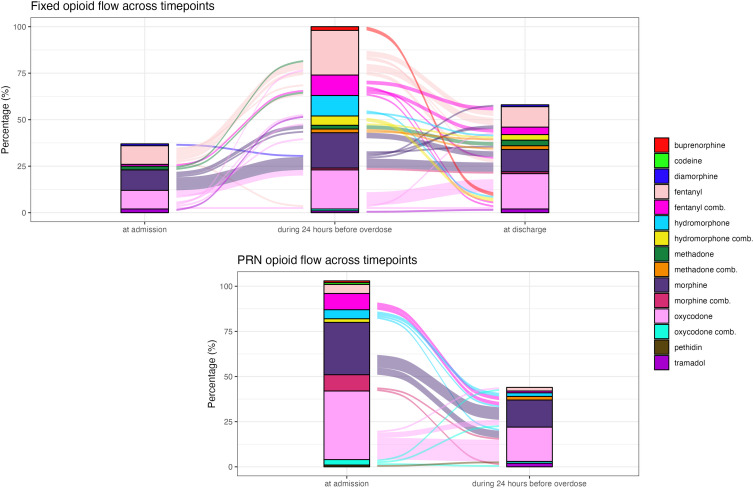
Sankey plot of opioid prescriptions at hospital admission, during the 24 hours before opioid overdose and at discharge. comb. = combination, indicating the respective opioid co-prescribed with one or more other opioids, with the named opioid providing the highest dose.

There were also changes in the opioid substances prescribed. Of the 38 patients receiving regular opioid therapy at admission, 12 (32%) were taking morphine, 11 (29%) fentanyl, 10 (26%) oxycodone, 2 (5%) methadone, 2 (5%) tramadol and 1 (3%) diamorphine. Among the 100 patients taking fixed doses of opioids before their overdose, the most frequently prescribed were fentanyl (35, 35%), oxycodone (30, 30%), morphine (25, 25%) and hydromorphone (22, 22%). Of the 58 patients taking fixed opioid doses at discharge, 22 (38%) were taking oxycodone, 17 (29%) morphine and 15 (26%) fentanyl. See Figure 3 for a graphical overview of changes in regular and PRN opioid prescriptions.

### Preventability

Following the Schumock and Thornton preventability criteria,^21^ we found 82 (68%) cases of opioid overdose to be potentially preventable The reasons for preventability, of which multiple could apply, involved drug–drug interactions in 73 (89%) cases, inappropriate dosing in 15 (18%) and previous documented opioid overdoses in 4 (5%). Drug–drug interactions were most commonly pharmacodynamic in nature, meaning that multiple sedative drugs were combined. A key contributor to this category was the concurrent prescription of multiple opioids as regular medications (eg. morphine and oxycodone, both prescribed for continuous use). Inappropriate dosing was related to excessive doses, particularly in situations of renal insufficiency or older age.

### Outcome of the Hospitalisation

Seventeen (14%) patients died during their hospitalisation, but their deaths were not directly linked to opioid overdoses. Of the remaining 104 patients, 30 (29%) were discharged to a rehabilitation clinic, 28 (27%) to another hospital, 25 (24%) to their home, 18 (17%) to a nursing home and 3 (3%) to different institutions.

## Discussion

This ten-year retrospective case-series analysis estimated a yearly incidence of 11.0 naloxone treatments per 10,000 inpatients. Patients received a median of 73 MME in the 24 hours before their opioid overdose and took more sedative drugs than at hospital admission. Drug use patterns changed from admission to overdose and on to hospital discharge. Importantly, more patients were taking opioids at discharge (68%) than at admission (56%); however, their median doses were lower (15 vs 29.4 MME. We deemed most of the opioid overdoses identified (68%) to have been potentially preventable, primarily due to drug–drug interactions.

We also estimated a yearly incidence of 52.7 naloxone treatments per 10,000 inpatients receiving opioids. A 2024 review reported a wide range of naloxone incidences, from 3.65–53.28 per 10,000 individuals; however, the review had only included postoperative patients.^11^ One study including medical patients reported an incidence of 11.5 naloxone treatments per 10,000 inpatients.^12^ Another study evaluating patients treated with naloxone across 12 hospitals found an incidence of 108.5 per 10,000 inpatients receiving opioids.^13^ Given there is limited information on naloxone treatment incidences, we found similar^12^ or lower incidences.^11^,^13^

Yearly incidences fluctuated considerably: they increased from 2014 to 2016, with the following years varying around the new, higher trend line. Because our case series was retrospective, we were unable to identify drivers of this increase. However, we should note that overall opioid sales in Switzerland increased from 2006 to 2013.^18^ Another national study reported increasing opioid consumption up until 2019, with the most pronounced increase occurring up to 2016.^25^ The same study evaluated calls to the national poison centre and found that from 2000 to 2019, rates had increased from 1.4 to 3.89 calls per 100,000 inhabitants.^25^ Considering the significant fluctuations, the observed increase in naloxone treatments could be partly linked to increased opioid use. Yearly naloxone use per 10,000 inpatients on opioids in our institution showed no such trend. This could indicate that the increased incidence of iatrogenic opioid overdoses was driven by increased prescriptions of opioids.

Our institution’s patients received a median of 73 MME in the 24 hours before their opioid overdose. Many studies have reported 100 MME per day as the opioid dose threshold significantly associated with overdoses.^26–28^ However, a 2018 systematic review found considerable variability in reported opioid doses, calculating that 68% of patients experiencing an overdose had received < 100 MME. Additionally, doses above 20 MME had been associated with opioid overdoses and doses above 50 MME with fatalities.^29^ This threshold has been adopted by policymakers: the American Center for Disease Control and Prevention advises clinicians to pause and reassess risks and benefits when prescribing opioid doses higher than 50 MME.^24^ Thus, the median opioid dose identified in our study was within the ranges reported in the literature. Nevertheless, the findings seem to be very heterogeneous.

We estimated that the majority (68%) of the opioid overdoses identified in our institution had been potentially preventable A 2010 chart review study in six community hospitals found that 67% of ADEs related to narcotic analgesics were preventable; however, this was based on only 30 cases, and the authors also used slightly different definitions of what they considered preventable.^30^ An older study from 1992, performed in two acute care teaching hospitals, found that 64% of opioid overdoses had been preventable, but this study only identified 22 cases and had used slightly different preventability criteria.^31^ Even though few studies have reported on the preventability of opioid overdoses, and despite their different settings and time periods, we found a similar rate of preventable opioid overdoses.

The main reason why we considered many opioid overdoses to be preventable was drug–drug interactions. Specifically, we found many cases of patients receiving high sedative loads in addition to simultaneous, around-the-clock dosing with different opioids. It is well established that combining opioids and other sedatives increases the risk of an opioid overdose.^32^ This has resulted in a warning issued by the United States Federal Drug Administration.^33^ Similarly, it is well recognised, and reflected in prominent guidelines, that simultaneously combining multiple opioids around the clock has no proven benefits but increases the risk of an overdose.^24^ It is of note that this is in contrast to situations where a regular opioid treatment is unavailable as a PRN medication and has to be combined with a different opioid (eg. a fentanyl transdermal therapeutic system combined with oxycodone rapid release capsules). In conclusion, the reasons we used to evaluate potentially preventable opioid overdoses were based on solid evidence and reflected in relevant guidelines.

While none of the iatrogenic opioid overdoses we detected were lethal, they may still have harmed the patients affected. There is evidence that opioid overdoses are associated with longer hospital stays^9^ and they may also lead to hospital readmissions.^10^ Cumulatively, these outcomes lead to higher healthcare costs.^8^ Lacking a reference group, our study could not determine the risk factors for iatrogenic opioid overdoses. Quantitatively, however, the cases we identified had a long median hospital length of stay (20 days). Our patients had a median age of 67 and multiple chronic diseases, indicating a complex population. Thus, considering our retrospective case study design, we found that patients requiring naloxone had long hospitalisations and were old and multimorbid, consistent with previous studies. Given these findings, suitable measures to prevent such opioid overdoses should be evaluated.

Several measures could be implemented to reduce the risk of opioid overdoses. Primary prevention of iatrogenic opioid overdoses could include reducing opioid prescriptions overall, regular risk evaluations for opioid overdoses, and professional and patient education.^34^ More specifically, suitable trigger tools could be used to detect and flag high-risk drug combinations involving opioids.^35^ However, alert fatigue must be considered when such tools warn prescribers directly.^36^ One way to circumvent this would be preliminary, trigger-based clinical pharmacy reviews of patients treated with high-risk opioid combinations.^35^ A trigger tool for this was recently developed, validated^37^ and implemented in a multimodal, clinical pharmacy intervention.^38^ Secondary prevention measures might consist of monitoring prescriptions (opioid and naloxone use) and the continued evaluation of the benefit–risk ratios of prescribed opioids.^34^ Tertiary prevention measures could include prescribing intranasal naloxone to high-risk patients and the treatment of opioid use disorder.^34^ In conclusion, opioid stewardship measures that could increase patient safety do exist.

Lastly, further research is needed into the safety of naloxone. In particular, the prevalence of opioid withdrawal symptoms following naloxone administration requires further investigation.

## Limitations

The present study had some limitations. First, the lack of a control group affects how we can interpret its results. We cannot draw any firm conclusions on the risk factors of opioid overdose. Second, we relied on chart reviews to extract our information, and we had no way of ensuring that the clinical records were complete. Third, we did not have access to opioid overdose data from intensive or intermediate care units and operating rooms, which reduces the generalisability to the entire hospital population of our findings.

## Conclusion

This ten-year retrospective case series analysis found approximately 11 opioid overdoses treated with naloxone per 10,000 inpatients per year, which is comparable with other studies. We rated a majority (68%) of overdoses as having been potentially preventable, mainly because they had involved the concurrent use of multiple opioids or drug combinations with other sedatives. We believe that these findings warrant more research into the need for concrete measures to prevent opioid overdoses and increase patient safety, such as improved professional education or modern prescribing trigger tools.

## Data Availability

The dataset analysed in this study are available from the corresponding author upon reasonable request.
